# Molecular imaging phenotyping for selecting and monitoring radioligand therapy of neuroendocrine neoplasms

**DOI:** 10.1186/s40644-022-00465-3

**Published:** 2022-06-03

**Authors:** Amir Iravani, Ashwin Singh Parihar, Timothy Akhurst, Rodney J. Hicks

**Affiliations:** 1 Mallinckrodt Institute of Radiology, Washington University School of Medicine, 510 S Kingshighway Blvd, St. Louis, USA; 2 Department of Cancer Imaging, Peter MacCallum Cancer Centre, 305 Grattan Street, Melbourne, Australia; 3 The Sir Peter MacCallum Department of Oncology, University of Melbourne, 305 Grattan Street, Melbourne, Australia; 4 The Department of Medicine, St Vincent’s Hospital, University of Melbourne, Melbourne, Australia; 5 The Central Clinical School, Alfred Hospital, Monash University, Melbourne, Australia

**Keywords:** Neuroendocrine tumours, Molecular imaging, Somatostatin receptor, Peptide receptor radionuclide therapy, Radioligand therapy, Positron emission tomography

## Abstract

Neuroendocrine neoplasia (NEN) is an umbrella term that includes a widely heterogeneous disease group including well-differentiated neuroendocrine tumours (NETs), and aggressive neuroendocrine carcinomas (NECs). The site of origin of the NENs is linked to the intrinsic tumour biology and is predictive of the disease course. It is understood that NENs demonstrate significant biologic heterogeneity which ultimately translates to widely varying clinical presentations, disease course and prognosis. Thus, significant emphasis is laid on the pre-therapy evaluation of markers that can help predict tumour behavior and dynamically monitors the response during and after treatment. Most well-differentiated NENs express somatostatin receptors (SSTRs) which make them appropriate for peptide receptor radionuclide therapy (PRRT). However, the treatment outcomes of PRRT depend heavily on the adequacy of patient selection by molecular imaging phenotyping not only utilizing pre-treatment SSTR PET but ^18^F-Fluorodeoxyglucose (^18^F-FDG) PET to provide insights into the intra- or inter-tumoural heterogeneity of the metastatic disease. Molecular imaging phenotyping may go beyond patient selection and provide useful information during and post-treatment for monitoring of temporal heterogeneity of the disease and dynamically risk-stratify patients. In addition, advances in the understanding of genomic-phenotypic classifications of pheochromocytomas and paragangliomas led to an archetypical example in precision medicine by utilizing molecular imaging phenotyping to guide radioligand therapy. Novel non-SSTR based peptide receptors have also been explored diagnostically and therapeutically to overcome the tumour heterogeneity. In this paper, we review the current molecular imaging modalities that are being utilized for the characterization of the NENs with special emphasis on their role in patient selection for radioligand therapy.

## Introduction

Neuroendocrine neoplasms (NENs) represent a group of rare malignancies that originate from the secretory cells of the neuroendocrine system. NEN is an umbrella term that includes the well-differentiated neuroendocrine tumours (NETs), and the aggressive neuroendocrine carcinomas (NECs). The site of origin of the NENs is linked to the intrinsic tumour biology and is predictive of the disease course [1]. Based on their production of biogenic amines and vasoactive substances leading to distinct clinical symptoms, these tumours can be categorised as functioning or non-functioning. Further, for a given site, the NENs can be classified based on their histologic grade, and tumour differentiation. The various combinations of these parameters produce a wide spectrum of tumour biology ranging from well-differentiated, relatively indolent, localized tumours to poorly differentiated, aggressive and commonly metastatic carcinomas. Importantly, metastatic disease is not necessarily an indicator of high-grade tumour biology in this disease as many low-grade, well-differentiated NET also present with disseminated disease.

It is understood that NENs demonstrate significant biologic heterogeneity which ultimately translates to widely varying clinical presentations, disease course and prognosis. Thus, significant emphasis is laid on the pre-therapy evaluation of markers that can help predict tumour behavior and the appropriate selection of choice of therapy. These broadly include assessment of pathology (including Ki67 immunohistochemistry (IHC) or mitotic count as markers of proliferation, cellular differentiation, IHC for specific hormones or cell surface antigens), serum biomarkers, molecular genetics (in both sporadic and familial tumours), clinical presentation and imaging (conventional and molecular imaging).

Imaging plays a vital role in the diagnosis, staging and therapeutic monitoring of NEN [2]. While surgical planning is critically dependent on the detailed anatomical evaluation provided by diagnostic multiphase CT or MRI, selection and monitoring of systemic therapies are increasingly dependent on the use of molecular imaging, particularly using somatostatin analogs. Most low-grade and well-differentiated NENs express somatostatin receptors (SSTRs), which are G-protein coupled transmembrane receptors modulating cellular proliferative and secretory activity. SSTR sub-types 2, 3 and 5 are most commonly expressed on the NEN cells with the dominant subtype 2 being primarily targeted for molecular imaging using positron emission tomography (PET) or single-photon emission computed tomography (SPECT) [3]. SSTR PET using ^68^Ga- or ^64^Cu- labeled somatostatin analogs (DOTANOC, DOTATATE, DOTATOC, SARTATE) has superior diagnostic performance in comparison to SPECT with ^111^In-Pentetreotide (Octreoscan), with the former being the modality of choice for functional imaging of the NETs [4, 5]. ^18^F-Fluorodeoxyglucose (^18^F-FDG), the ubiquitous radiotracer for oncologic PET imaging has a limited role in the detection of low-grade, well-differentiated NENs. However, it can provide useful insights into the intra- or inter-tumoural heterogeneity in a patient with metastatic disease. Nevertheless, there is evidence that there are subgroups of NEN that lack SSTRs [6]. These are primarily higher grade NENs, which have imaging features similar to other aggressive cancers in having augmented glycolytic metabolism. Accordingly, ^18^F-FDG can provide complementary information, particularly by the detection of more aggressive and poorly-differentiated tumour foci, that can co-exist in patients with the otherwise low-grade disease [4]. Alternative molecular targets are available for specific subcategories of NEN. For example, ^123^I/^131^I-meta-iodobenzylguanidine (^123^I/^131^I-MIBG) has been utilized for imaging tumours of neuroendocrine origin, especially pheochromocytoma, paragangliomas and pediatric neuroblastic tumours [7]. Additionally, other radiopharmaceuticals such as ^18^F-Fluoro-L-Dihydroxyphenylalanine (^18^F-DOPA), ^68^Ga-Exendin, and other experimental radiotracers are being utilized for capturing different metabolic pathways or differential receptor expression in the NENs [8–10].

Selection of the most appropriate treatment for NEN depends on an accurate definition of disease extent and biological characteristics. Independent of grade, surgery is curative for localized NETs. Unfortunately, over 40% of patients present with either advanced loco-regional or distant metastases and are already unsuitable for curative resection at diagnosis but may benefit from debulking surgery [11]. For such cases, disease characterization becomes increasingly important with options ranging from observation to aggressive chemotherapy regimens.

For low-grade but functioning tumours, somatostatin analogs (SSAs) are the usual first-line treatment. These agents have also been shown to delay the progression of unresectable NETs [12, 13]. Despite these agents requiring expression SSTRs for efficacy, confirmation of their presence on molecular imaging is not currently mandated, primarily because of the near-ubiquitous SSTR expression in the majority of low-grade NENs. Nevertheless, the high cost and inconvenience of long-acting SSAs may warrant the characterization of SSTR expression, particularly in NETs of the lung, which can be functional but sometimes lack this target [14] or in patients in whom there is a failure to control hormonal symptoms or tumour growth.

The expression of SSTRs can also be leveraged therapeutically using SSAs labelled with particle-emitting radionuclides. Peptide receptor radionuclide therapy (PRRT), most widely available with ^177^Lu-DOTATATE, has emerged as a valuable treatment modality for metastatic NENs and showed superior outcomes over the standard-of-care in patients with inoperable or advanced and progressive midgut NETs in a phase-3 randomized controlled trial (NETTER-1) [15]. However, the treatment outcomes of PRRT depend heavily on the adequacy of patient selection, performed using a combination of imaging and non-imaging techniques, as highlighted previously [4, 16, 17].

In this paper, we review the current molecular imaging modalities that are being utilized for the characterization of the NENs with special emphasis on their role in patient selection for radioligand therapy and monitoring its efficacy.

## Pre-treatment molecular imaging phenotyping

### SSTR PET/CT

SSTR targeted imaging forms the principal basis for PRRT by documenting adequate tumoural SSTR expression, which is the therapeutic target. The Krenning score was developed to grade the degree of radiotracer avidity on ^111^In-pentetreotide scintigraphy and was later modified for use with SSTR PET [18]. The modified Krenning score is a 5-point visual scale that compares the lesional radiotracer avidity with that of the blood pool, liver and spleen. The grading is done as 0: no avidity (less than blood pool), 1: very low avidity (equivalent to blood pool), 2: avidity less than or equal to the liver but more than blood pool, 3: avidity higher than liver but less than the spleen, 4: avidity equal to or higher than the spleen. For lesions > 2 cm, the scores of 3–4 are deemed adequate for considering PRRT. However, for lesions smaller than 2 cm, the PET-based scoring might overestimate compared to the original Krenning score [4]. In principle, PRRT should not be considered in patients with the majority of lesions demonstrating no or low avidity on SSTR PET. Neither should the mere presence of SSTR expression in a lesion or a single voxel, assessed visually or based on standardised uptake value (SUV), be an indication for PRRT, which must always consider the clinical scenario while leveraging the unique advantage of whole-body assessment on SSTR PET in treatment selection.

The intrinsic heterogeneity of NENs is evident on SSTR PET with inter-and intra-lesional heterogeneity in the expression of the SSTRs. This was formally assessed in the context of PRRT in a retrospective review of 65 patients with WHO Grade 1–2 progressive NENs [19]. Intra-lesional heterogeneity was assessed as visually interpreted changes in the modified Krenning score across a single target lesion. A patient was categorized as heterogeneous if ≥ 50% of their target lesions were heterogeneous. If < 50% of target lesions were heterogeneous, the patient was categorized as homogeneous. Patients with heterogeneous SSTR expression had a significantly lower overall survival (OS) and time-to-progression. Additionally, heterogeneity was the only prognostic factor for OS in the multivariable analysis. Currently, there is no validated methodology to assess the intra-lesional heterogeneity while semiautomated approaches are in development with early promising results [20]. Ultimately, imaging has a finite resolution and the microscopic heterogeneity cannot adequately be captured at a cellular level within a tumour mass. It should be noted that microscopic heterogeneity is also recognised by pathologists and dealt with in histopathological specimens by scoring “hot spots” for immunohistochemical parameters like Ki-67.

### ^18^F-FDG PET/CT

^18^F-FDG is a radioactive analog of glucose, preferentially localizing in cells with high glycolytic rates. ^18^F-FDG avidity reflects the viable tumour cells with high glucose metabolism, and commonly correlates with tumour aggressiveness, i.e. highly aggressive malignancies show a higher uptake of ^18^F-FDG in comparison to the relatively indolent tumours [21]. It is thus understandable that most low-grade, well-differentiated NETs do not show high ^18^F-FDG avidity and should be preferentially imaged using SSTR PET for staging [22]. ^18^F-FDG PET/CT is more useful in staging patients with high grade, and aggressive NENs which frequently lose SSTR expression.

In a prospective series of 98 patients with primary NENs of gastro-entero-pancreatic (GEP) or pulmonary origin, 57 (58%) had an ^18^F-FDG avid disease on PET/CT. Disease positivity on ^18^F-FDG PET/CT was associated with a significantly higher risk of mortality (hazard ratio 10.3; 95% CI: 1.3–78.9). The disease positivity on ^18^F-FDG PET was higher in patients with increasing histologic grade (40% in patients with World Health Organization (WHO) Grade 1 and 93% in Grade 3). Additionally, in a multivariable analysis including SUVmax, Ki67 and chromogranin-A levels, only SUVmax > 3 on ^18^F-FDG PET/CT was a predictor of progression-free survival (PFS) [23]. Similar findings were reported in a prospective series of 38 patients with metastatic GEP NETs. The OS was significantly longer in patients with a negative ^18^F-FDG PET than in those with a positive ^18^F-FDG PET [24].

In the context of PRRT, ^18^F-FDG PET/CT is shown to be an independent predictor of treatment outcomes. In a retrospective review of 495 patients with NENs who underwent PRRT (with single-agent or combination ^177^Lu-DOTATATE/ ^90^Y-DOTATOC), the OS and PFS were significantly longer in patients with a negative ^18^F-FDG PET, compared to those with a positive ^18^F-FDG PET at baseline [25].

**Fig. 1:**
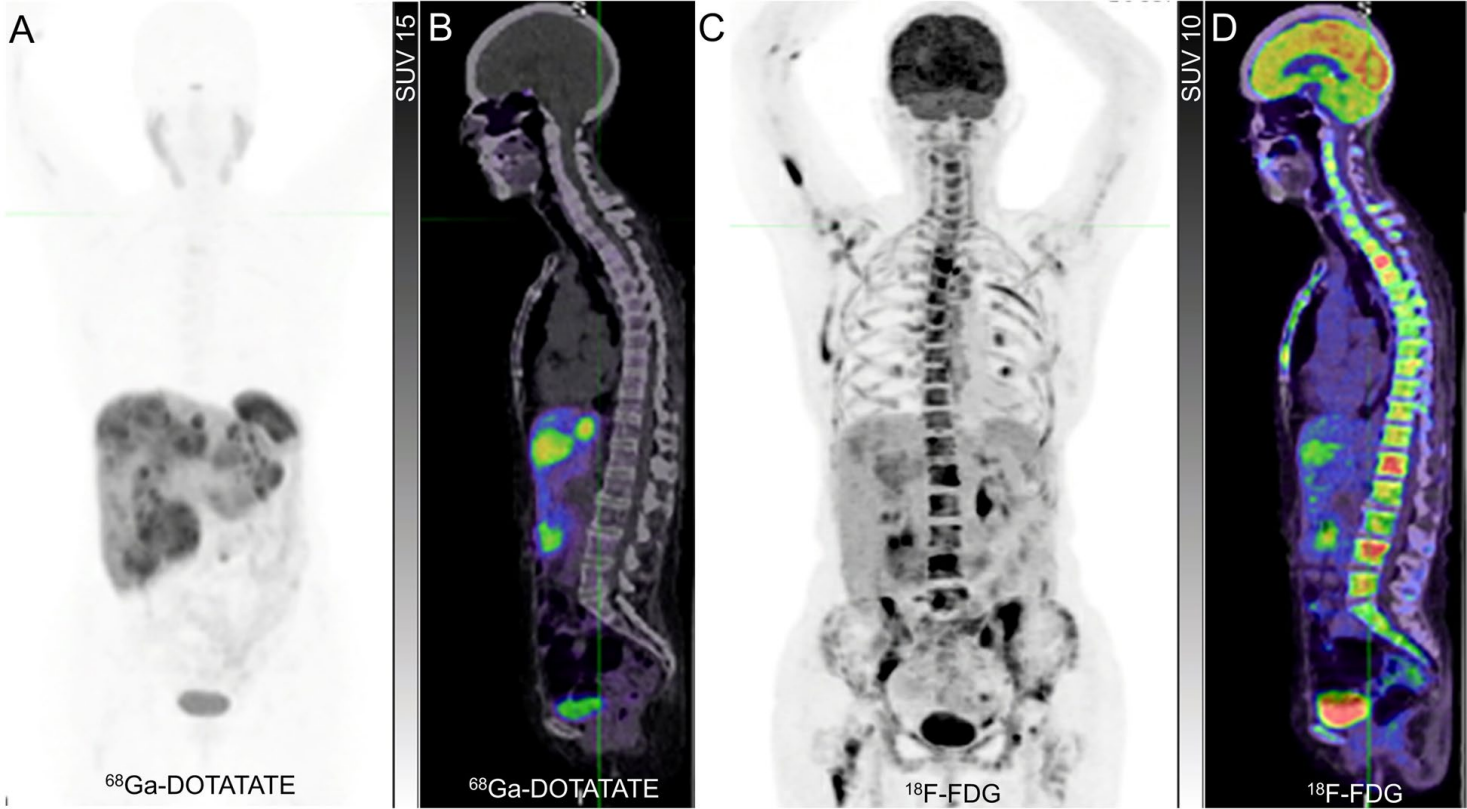
Discordant ^68^Ga-DOTATATE and ^18^F-FDG PET due to a second malignancy. A patient with a small intestinal NET, initially of G1 with Ki-67 2% but subsequently converted to G3 with Ki-67 30% based on later liver biopsy, with contemporaneous ^68^Ga-DOTATATE PET (**A**) and ^18^F-FDG PET (**C**) demonstrating concordant tracer avid known liver disease but extensive and heterogeneous ^18^F-FDG-avid/non-^68^Ga-DOTATATE avid in the axial and proximal appendicular skeleton, also demonstrated on sagittal fused PET/CT (**B** and **D**). Given the absence of skeletal uptake on the ^68^Ga-DOTATATE PET and high ^18^F-FDG uptake, bone marrow biopsy was performed demonstrating excess myeloid blasts consistent with early acute myeloid leukemia. Interestingly, blood counts were not significantly deranged at that time. No evidence of NET metastasis was identified on the bone marrow trephine

The mechanism of high ^18^F-FDG avidity in patients with WHO Grade 1 NET requires consideration as this is out of keeping with the expected biology of such tumours. In some cases, this is likely to reflect a pathological sampling error related to heterogeneous grades of the disease being simultaneously present. In other cases, it might reflect the loss of differentiation between the initial biopsy and subsequent imaging. Given the generally indolent nature of NEN, several years may elapse between biopsy confirmation of a primary NEN and the development or progression of metastatic disease requiring consideration of active treatment. Accordingly, the initial biopsy may no longer be representative of the current grade of disease. Demonstration of unexpected ^18^F-FDG avidity in a previously low-grade NEN may warrant targeted histologic re-evaluation. Other reasons for high ^18^F-FDG avidity may include biological processes that increase tissue glycolysis, including hypoxia and inflammation. The possibility of a synchronous or metachronous malignancy should also be considered (Fig. 1).

### Dual tracer (SSTR and.^18^F-FDG PET) imaging

**Fig. 2:**
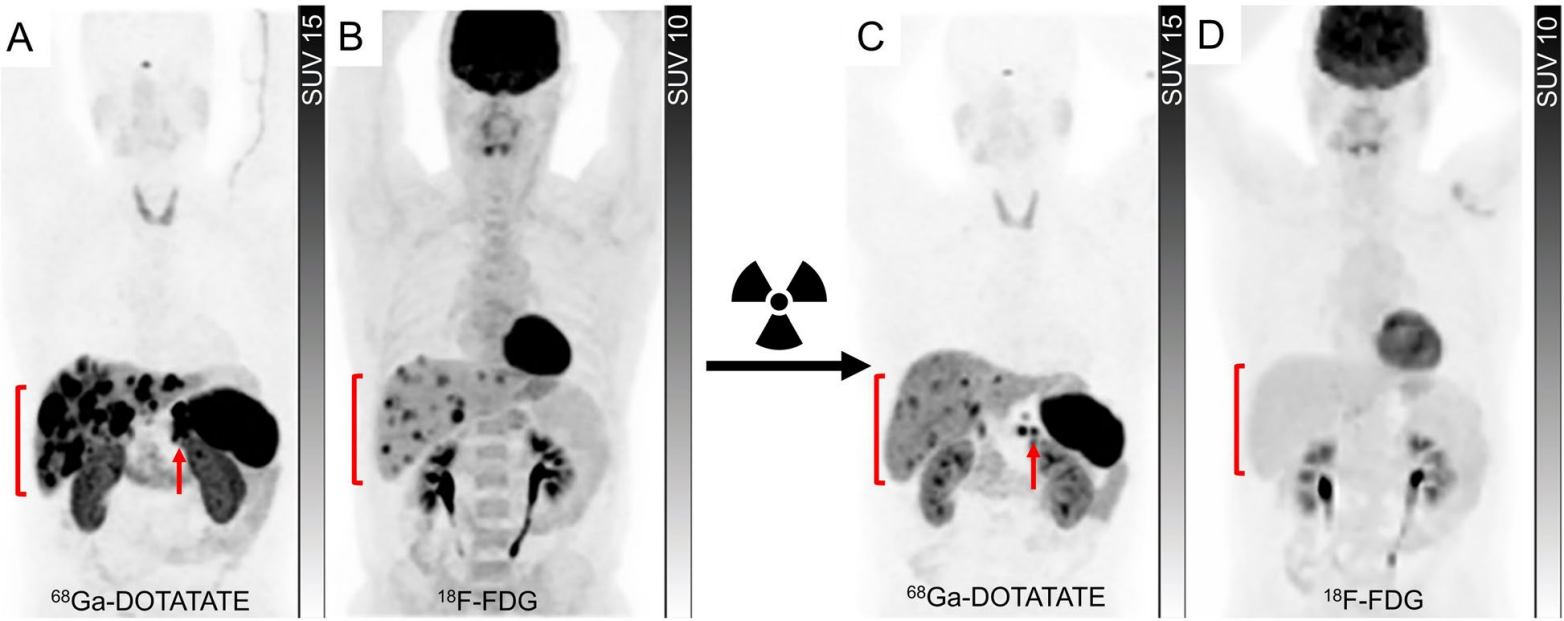
Monitoring of PRRT using dual tracer approach. A patient with pancreatic NET (G3, Ki-67 30%) with ^68^Ga-DOTATATE PET (**A**) showed a large volume of intensely tracer-avid hepatic metastases (bracket) and a pancreatic mass (arrow). Contemporaneous ^18^F-FDG PET (**B**) showed some of the hepatic lesions are moderate to intensely avid (bracket) but with no ^18^F-FDG-avid/non-^68^Ga-DOTATATE-avid disease; hence the patient was deemed suitable for PRRT. After 4 cycles of ^177^Lu-DOTATATE, There was a significant reduction in the tumour burden on ^68^Ga-DOTATATE PET (**C**, bracket and arrow) and a complete metabolic response on ^18^F-FDG PET (**D**, bracket). Despite persisting SSTR-expressing disease, the loss of ^18^F-FDG avidity suggested an improved prognosis. The patient remained well more than 10-years after initial PRRT despite requiring additional PRRT for relapsing SSTR-expressing disease

While SSTR PET is the primary molecular imaging technique for WHO Grade 1 and 2 NET and ^18^F-FDG PET is reserved for patients with WHO Grade 3 NENs and NECs, in which SSTR expression is either low or absent, ^18^F-FDG PET can provide complementary information by detecting the aggressive, poorly differentiated, and high-grade disease that may co-exist with the low-grade disease in a patient. The combination of high SSTR and low ^18^F-FDG avidity on PET increases the likelihood of an indolent natural history and the decision to perform PRRT should be based on either uncontrolled symptoms or demonstrable disease progression on long-acting SSAs. When both scans are positive but no lesions are lacking SSTR expression, PRRT remains a therapeutic option but the need for the institution of therapy becomes more acute due to the prognostic implications of ^18^F-FDG avidity detailed above. As indicated, the combined results from SSTR and ^18^F-FDG PET can help in the biopsy planning, as the lesion with the highest ratio of ^18^F-FDG to SSTR avidity is likely to represent the most aggressive disease that will eventually determine survival outcomes. Although ^18^F-FDG PET has demonstrated the most significant impact on clinical management in patients with WHO Grade 2 and 3 NETs [26], a positive ^18^F-FDG PET has been shown to significantly lower the survival outcomes even in patients with metastatic WHO Grade 1 NET and a positive SSTR PET [24]. Despite the tendency to lose SSTR with increasing grade, ^18^F-FDG avid WHO Grade 3 NET and NEC that continue to express sufficient SSTRs to warrant PRRT can have a high objective response rate to this treatment, presumably related to increased radiosensitivity of actively proliferating cells [27]. Interestingly, the ^18^F-FDG avid elements of the disease may be more responsive to PRRT as reflected by a more marked response on ^18^F-FDG than on SSTR PET monitoring (Fig. 2). Despite a higher response rate, the durability of response to PRRT tends to be shorter in G3 NEN. This is akin the higher ORRs but shorter median OS of G3 NEN with a Ki-67 > 55% than < 55% with carboplatin/etoposide chemotherapy [28]. It appears that the disease positivity on SSTR PET does not obviate the need for performing an ^18^F-FDG PET/CT, as the latter bears greater prognostic and predictive utility, especially in the setting of PRRT.

Several grading systems have been devised to classify the patients into multiple distinct groups by incorporating both SSTR PET and ^18^F-FDG PET imaging with therapeutic or prognostic implications [14, 29, 30]. The NETPET categorical six-point scale (P0-P5) is applied to the single representative NEN lesion that has the maximum avidity on ^18^F-FDG PET relative to that on SSTR PET [29]. This single-lesion assessment aims to capture the most aggressive tumour phenotype within the overall disease burden of a patient. P1 (SSTR PET positive, ^18^F-FDG negative) represents the most favorable outcome whereas P5 (SSTR PET negative, ^18^F-FDG positive) suggests the worst outcomes and unlikely benefit from PRRT (Table 1). P2 to P4 categories have an additional descriptor ‘a’ (one or two lesions) or ‘b’ (three or more lesions). In a retrospective review of 62 patients with NENs, the NETPET grouping was significantly correlated with the WHO grade and OS [29]. Others have used more simplified approaches. Karfis et al., classified the patients into three groups, C1: all lesions ^18^F-FDG negative/^68^Ga-DOTATATE positive, C2: patients with one or more ^18^F-FDG positive lesions, all of them ^68^Ga-DOTATATE positive, C3: patients with one or more ^18^F-FDG positive lesions, at least one of them ^68^Ga-DOTATATE negative (Table 1) [30]. This three-scale molecular imaging phenotyping was correlated with the PFS of patients with GEP NENs. Zidan et al. classified the patients with pulmonary NENs into 4 molecular imaging groups and utilized this system to assess their suitability for PRRT [14]. While in the abovementioned two systems the background was used as the reference for positivity of the scan, in this system the liver, which is a common reference for the patient selection for PRRT, was used as the threshold for positivity on either ^18^F-FDG or ^68^Ga-DOTATATE PET. The patients were grouped to molecular imaging phenotype 1: all lesions negative on both scans; 2: all lesions ^68^Ga-DOTATATE positive/^18^F-FDG negative; 3: all lesions ^68^Ga-DOTATATE positive but some or all also ^18^F-FDG positive and 4: any ^68^Ga-DOTATATE negative/^18^F-FDG positive lesions (Table 1). Using this grading system almost half of the patients were deemed unsuitable (groups 1 and 4) for PRRT.

**Table 1:** Dual tracer molecular imaging grading scales of neuroendocrine tumours

Study	Reference for positive lesion	Group	SSTR PET	^18^F-FDG PET	Suitability for PRRT
Chan et al. [29] (NETPET)	> Background (Modified Krenning score 2–4)	P0	Negative	Negative	Not suitable
		P1	Positive	Negative	Probably suitable
		P2	Positive	Positive (avidity on ^18^F-FDG < SSTR)	Probably suitable
		P3	Positive	Positive (avidity on ^18^F-FDG = SSTR)	Probably suitable
		P4	Positive	Positive (avidity on ^18^F-FDG > SSTR)	Probably suitable if P4a^a^; Maybe suitable with additional therapy required if P4b
		P5	Negative	Positive	No
Karfis et al. [30]	> Background (Modified Krenning score 2–4)	C1	Positive (all lesions)	Negative (all lesions)	Probably suitable
		C2	Positive (all lesions)	Positive (≥ 1 lesion)	Probably suitable
		C3	Negative (≥ 1 lesion) ^b^	Positive (≥ 1 lesion)	Not suitable
Zidan et al. [14]	> Liver (Modified Krenning score 3–4)	1	Negative (all lesions)	Negative (all lesions	Not suitable
		2	Positive (all lesions)	Negative (all lesions)	Suitable
		3	Positive (all lesions)	Positive (≥ 1 lesion)	Suitable
		4	Negative (≥ 1 lesion)^b^	Positive (≥ 1 lesion)	Not suitable

^a^a – 1–2 lesions; b – 3 or more lesions showing the grade-specific pattern of avidity

^b^ At least one of the.^18^F-FDG avid lesions is SSTR PET negative

**Fig. 3:**
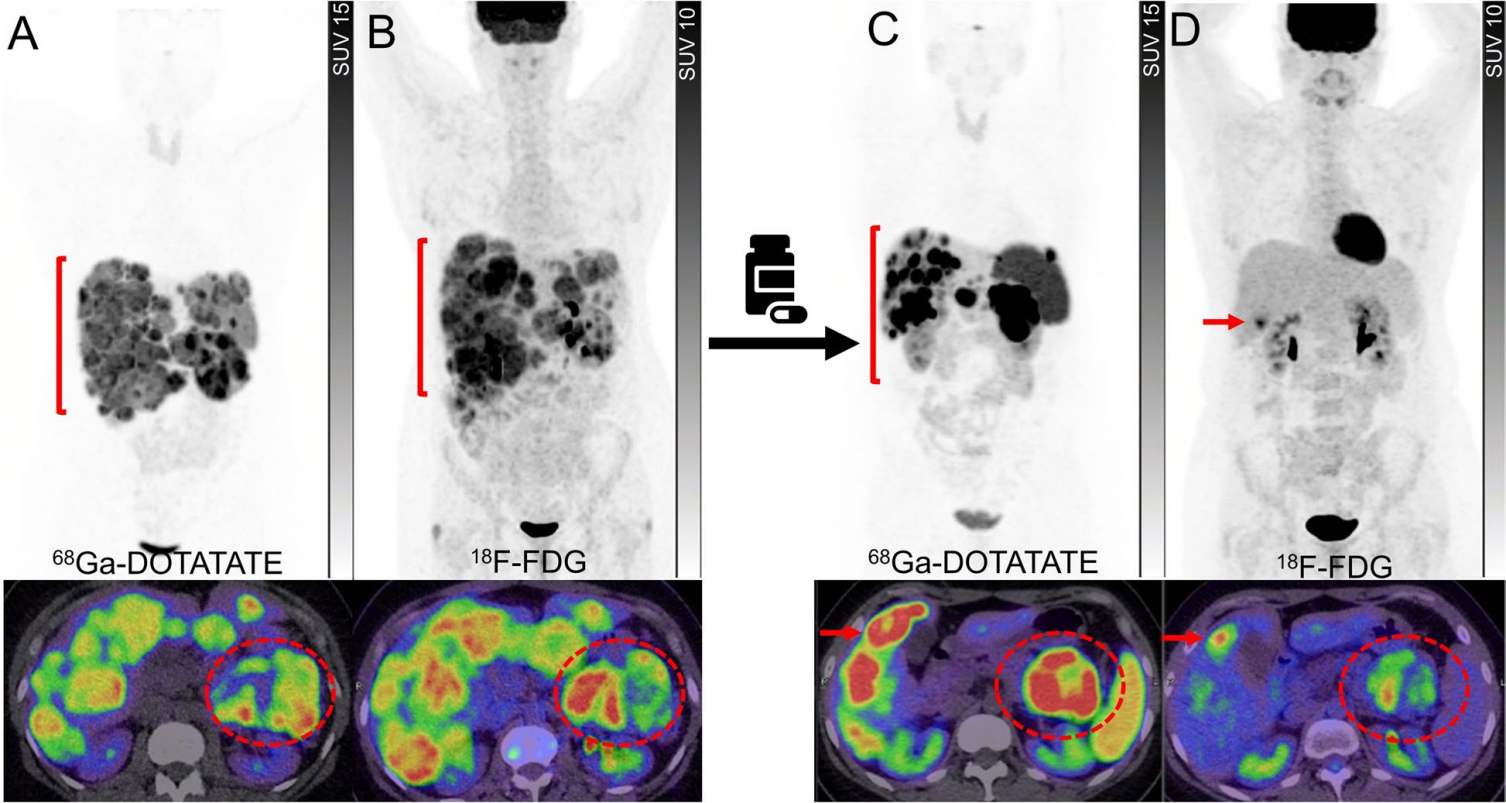
A patient with well-differentiated pancreatic NET (G3, Ki67 40%). ^68^Ga-DOTATATE PET (**A**) and ^18^F-FDG PET (**B**) showed heterogeneously tracer avid pancreatic mass with some areas (the medial component of the mass) demonstrating relatively higher ^18^F-FDG-avidity compared to ^68^Ga-DOTATATE avidity (circles). Large volume hepatic metastases were also noted with several areas demonstrating higher avidity on ^18^F-FDG PET (brackets). Due to high-grade pathology and a large-volume disease with some areas of heterogeneity on baseline scans, the patient was deemed not suitable for treatment with ^177^Lu-DOTATATE and therefore treated with chemotherapy. Restaging studies following 4 cycles of chemotherapy showed marked partial response on ^68^Ga-DOTATATE PET (**C**) with disease demonstrating more homogenous and higher tracer avidity (bracket). Contemporaneous ^18^F-FDG PET (**D**) showed marked partial response in the pancreas (circle) and resolution of metabolic activity in the liver except for one lesion which also demonstrated ^68^Ga-DOTATATE avidity (arrows). At this time point, the patient was deemed suitable for treatment with ^177^Lu-DOTATATE to consolidate the response to the prior treatment

**Fig. 4:**
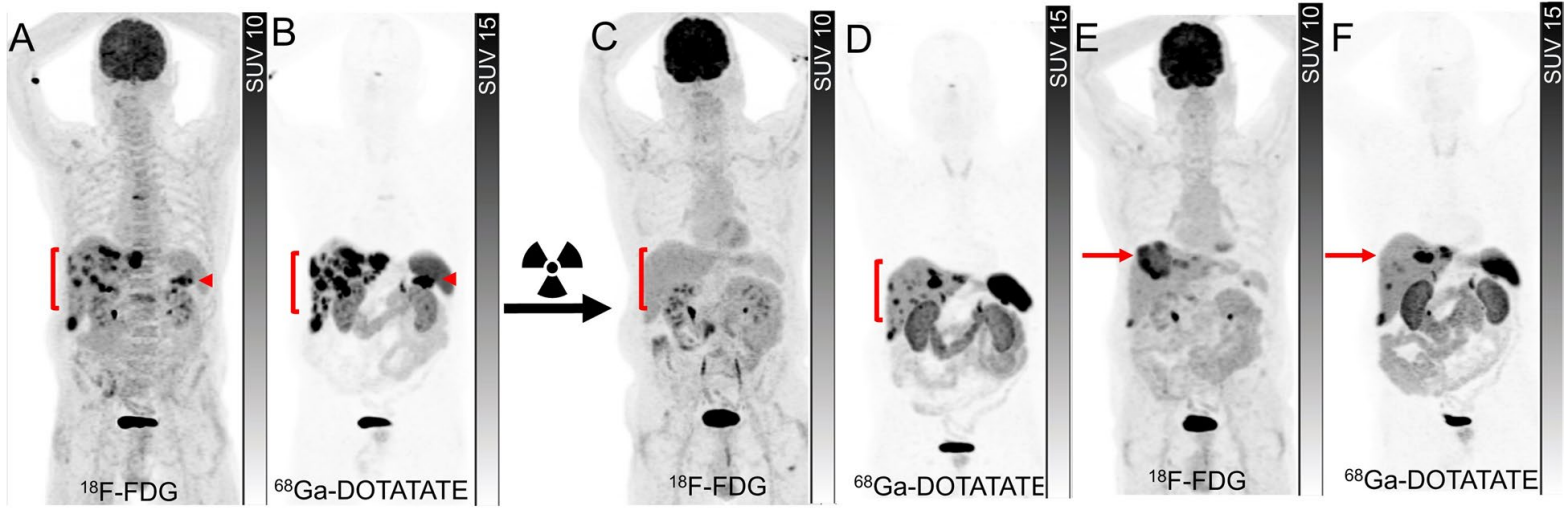
A patient with progressive metastatic pancreatic NET (G2, Ki67 10%) with glucagon hypersecretion with no prior treatments. ^18^F-FDG PET (**A**) and ^68^Ga-DOTATATE PET (**B**) showed a tracer avid lesion in the tail of the pancreas (arrowheads) and multiple hepatic metastases (brackets). As all lesions demonstrated intense ^68^Ga-DOTATATE uptake with no ^18^F-FDG-avid/non-^68^Ga-DOTATATE-avid disease, the disease was considered suitable for ^177^Lu-DOTATATE treatment. Restaging imaging 3 months after 4 cycles of ^177^Lu-DOTATATE showed complete metabolic response on ^18^F-FDG PET (**C**) and partial response on ^68^Ga-DOTATATE PET (**D**). Restaging ^18^F-FDG PET (**E**) and ^68^Ga-DOTATATE PET (**F**) 18 months later showed the development of an intensely ^18^F-FDG-avid/non-^68^Ga-DOTATATE-avid lesion in the dome of the liver (arrows), therefore the patient was deemed to be not suitable for retreatment with ^177^Lu-DOTATATE but considered for liver-directed therapy

Beyond the prognostic value of dual tracer imaging, the predictive value and guidance on the selection, rational sequencing or combination of treatment strategies and intensity of surveillance are of the additional value to these classifications (Figs. 3 and 4). For instance, the patients classified as having both SSTR and ^18^F-FDG positive disease may benefit from more intensified treatment with combination therapy using PRRT with chemotherapy either sequentially or concurrently to leverage the radiosensitizing capacity of many agents that are also active in NEN [31, 32]. There are several ongoing randomised phase II and III clinical trials comparing the efficacy and safety of chemotherapy or targeted therapies compared to PRRT in NENs (NCT03049189, NCT05247905, NCT04919226 and NCT04665739) but only some of those incorporated the dual tracer imaging in the inclusion criteria. While there would be a few years until the results of these studies become available, it remains important to go beyond the concept of comparative studies and refocus on individualizing the treatments to specific patient factors and biomarkers including dual tracer imaging.

## Special consideration in certain diseases

### Pulmonary NENs

**Fig. 5:**
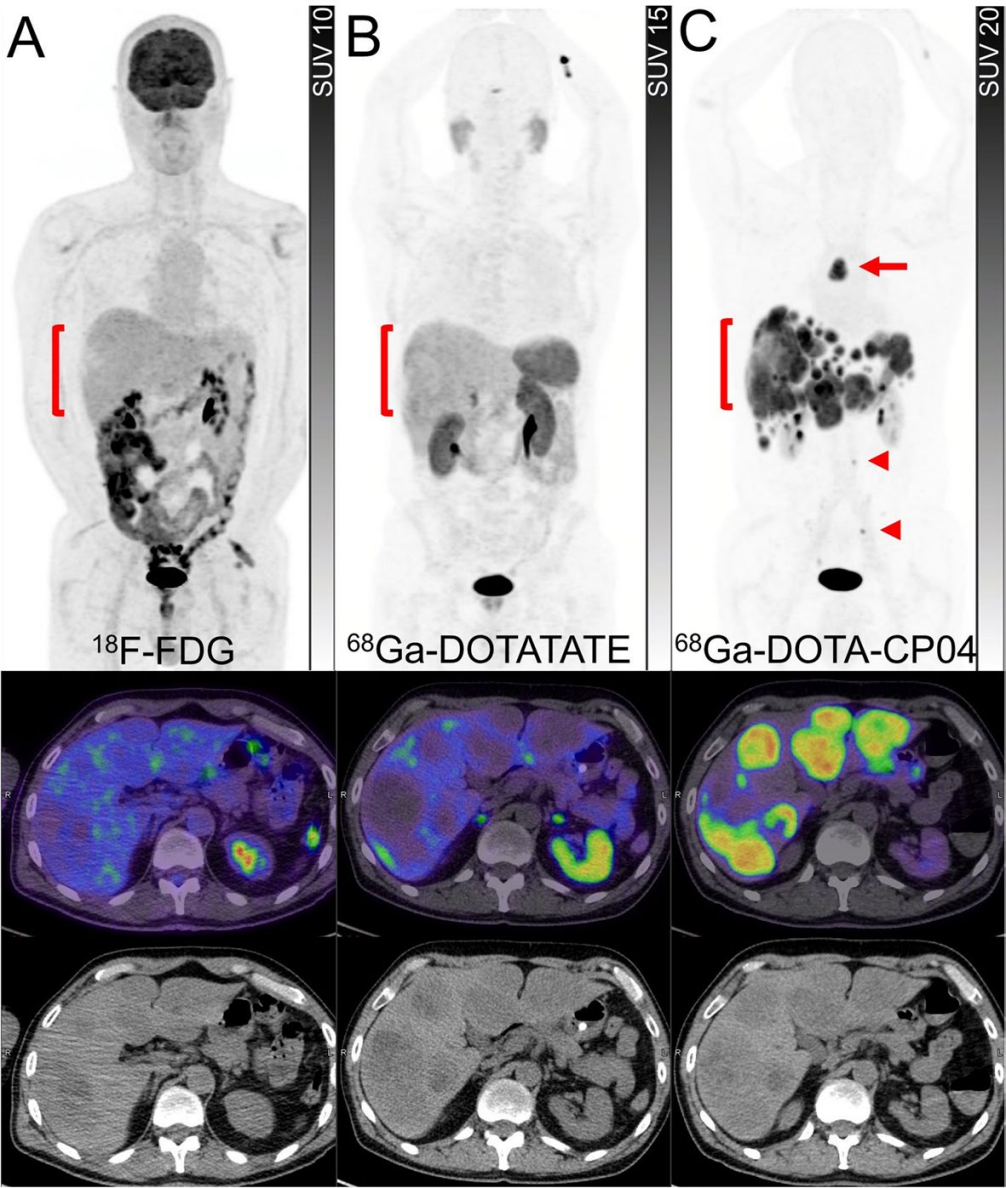
A patient with a progressive bronchial carcinoid tumour was referred for suitability for ^177^Lu-DOTATATE therapy. ^18^F-FDG PET, PET/CT and CT (**A**) showed non-avid hepatic metastases (brackets). ^68^Ga-DOTATATE PET, PET/CT and CT (**B**) demonstrated minimal tracer uptake in the periphery of hepatic metastases (brackets), indicating a lack of suitability for ^177^Lu-DOTATATE therapy. ^68^G-DOTA-CP04 (CCK-2 peptide receptor analog) PET, PET/CT and CT (**C**) showed intense uptake in all hepatic metastases (brackets) as well as a mediastinal nodal (arrow) and small bone metastases (arrowheads)

Pulmonary NENs are a very heterogeneous group of malignancies and are classified based on the degree of differentiation to either low-grade/well-differentiated tumours (typical and atypical pulmonary NETs) or high-grade/poorly differentiated carcinomas (large and small cell NECs). Wide variation in clinical behavior ranging from indolent tumours to highly aggressive tumours has posed challenges for standardization of care and made a case for personalized treatment strategies based on various factors [33]. Molecular imaging may provide some insight into the heterogeneity of the disease and the selection of patients for PRRT. A retrospective series of 18 patients with pulmonary NENs showed that all typical pulmonary NETs were positive on SSTR PET (11/11) while seven had a positive ^18^F-FDG PET. The ratio of SUVmax on FDG PET to SSTR PET was significantly greater in higher grade NENs in comparison to typical pulmonary NETs, consistent with the differences in tumour biology [34]. A prospective study of 31 patients with metastatic pulmonary NETs who underwent PRRT with ^177^Lu-DOTATATE showed a significantly higher incidence of ^18^F-FDG positive disease in patients with atypical (80%) in comparison to those with typical pulmonary NETs (28%). However, ^18^F-FDG PET positivity was not correlated with median PFS following PRRT [35]. In a study of 56 patients with bronchial NET who underwent contemporaneous SSTR and ^18^F-FDG PET, wide inter-and intra-patient heterogeneity was noted. Almost half of the patients were not potentially suitable for PRRT, emphasizing the important role of dual tracer imaging in selecting patients with pulmonary NET [14]. Other targets, such as the cholecystokinin-2 receptor may be an alternative option for diagnosis or therapy in some patients (Fig. 5).

**Fig. 6:**
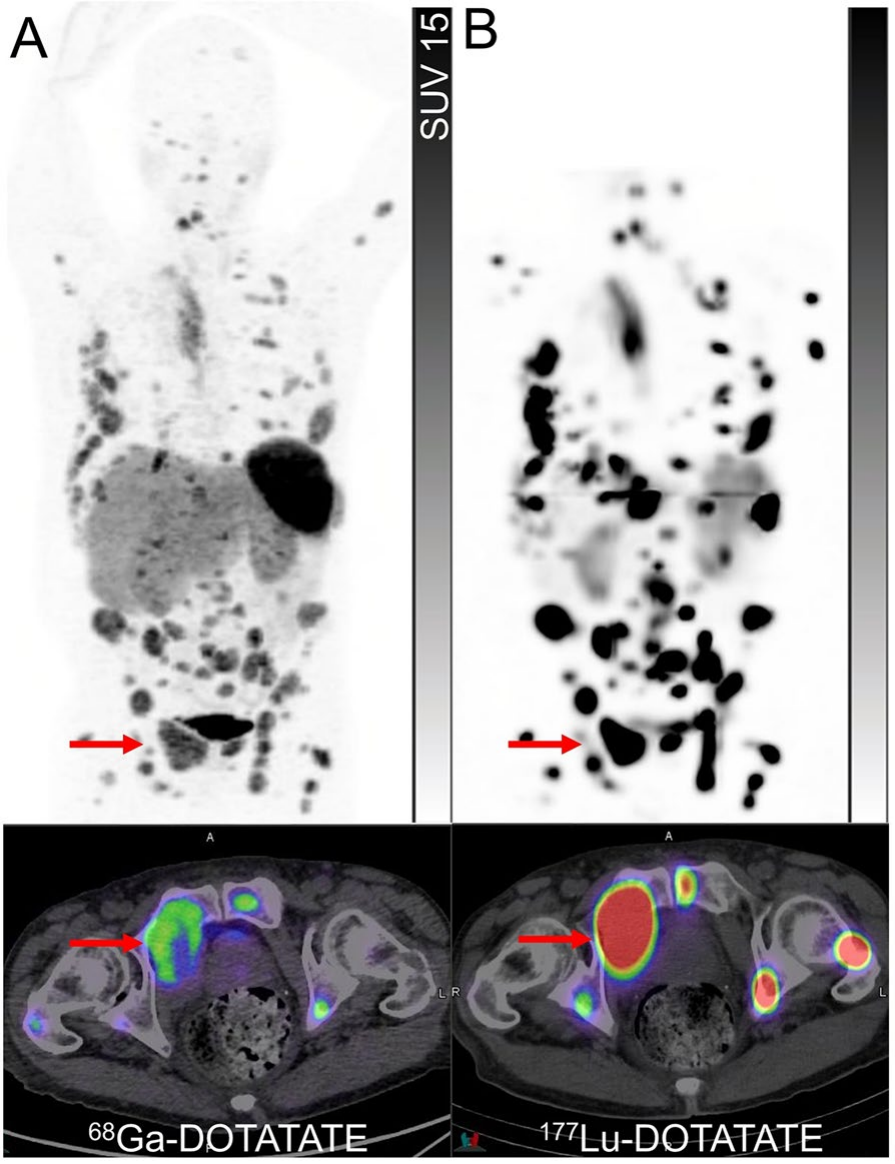
A patient with small cell lung cancer with progression after platinum-based chemotherapy and immune checkpoint monoclonal antibodies. ^68^Ga-DOTATATE PET and PET/CT (**A**) showed extensive moderately avid (Krenning score 3) osteolytic bone metastases (arrows). In the absence of other treatment options, the patient underwent ^177^Lu-DOTATATE, and post-treatment SPECT and SPECT/CT at 24 h (**B**) showed very high tracer retention (Krenning score 4), much higher than expected from baseline ^68^Ga-DOTATATE PET, in all sites of disease

Small-cell NECs represent the most aggressive spectrum of tumours with neuroendocrine features with a very high proliferation rate and dismal prognosis. Except for a small fraction of patients with durable responses to combination immune checkpoint monoclonal antibodies and platinum-based chemotherapy, response to currently available treatments is short-lived [36]. Although there is a trend toward decreased SSTR expression on IHC from well-differentiated NETs (typical and atypical bronchial carcinoids) to poorly differentiated NECs (large cell and small cell carcinomas), there remains a relevant SSTR expression in a subset of patients with NECs (Fig. 6) [37]. Preclinical evaluation has indicated that SSTR-expressing cell lines derived from small cell lung cancer can have a similar response to that achieved with carboplatin and etoposide chemotherapy and that the combination of both appeared synergistic, leading to clinical translation [38]. In a small study, 4/21 (19%) patients had ^68^Ga-DOTATATE positivity at all sites of disease, potentially suitable for treatment with ^177^Lu-DOTATATE [39]. In a phase I study of 9 patients with lung NENs (6 with small cell NECs) all patients underwent baseline ^68^Ga-DOTATATE and ^18^F-FDG PET and were treated with combination nivolumab and ^177^Lu-DOTATATE [40]. Although positivity on ^68^Ga-DOTATATE was considered as an inclusion criterion, patients without uptake were also included. Significant inter and intra-patient heterogeneity was noted between ^68^Ga-DOTATATE and ^18^F-FDG PET. The discrepancy is also noted between SSTR-2 IHC and ^68^Ga-DOTATATE, likely indicating inter or intralesional heterogeneity within the same individual or temporal heterogeneity as the archival tissue was used. Although the feasibility of the combination therapy was demonstrated, the only patient who achieved partial response had strong uptake on ^68^Ga-DOTATATE. Furthermore, on lesion-based analysis, none of the tumours without ^68^Ga-DOTATATE uptake exhibited size reduction. While ^177^Lu-DOTATATE appears to be an active therapy in a small subset of patients with this aggressive malignancy, the patient selection based on pre-treatment imaging remains of critical importance.

### Pheochromocytoma and paragangliomas

**Fig. 7:**
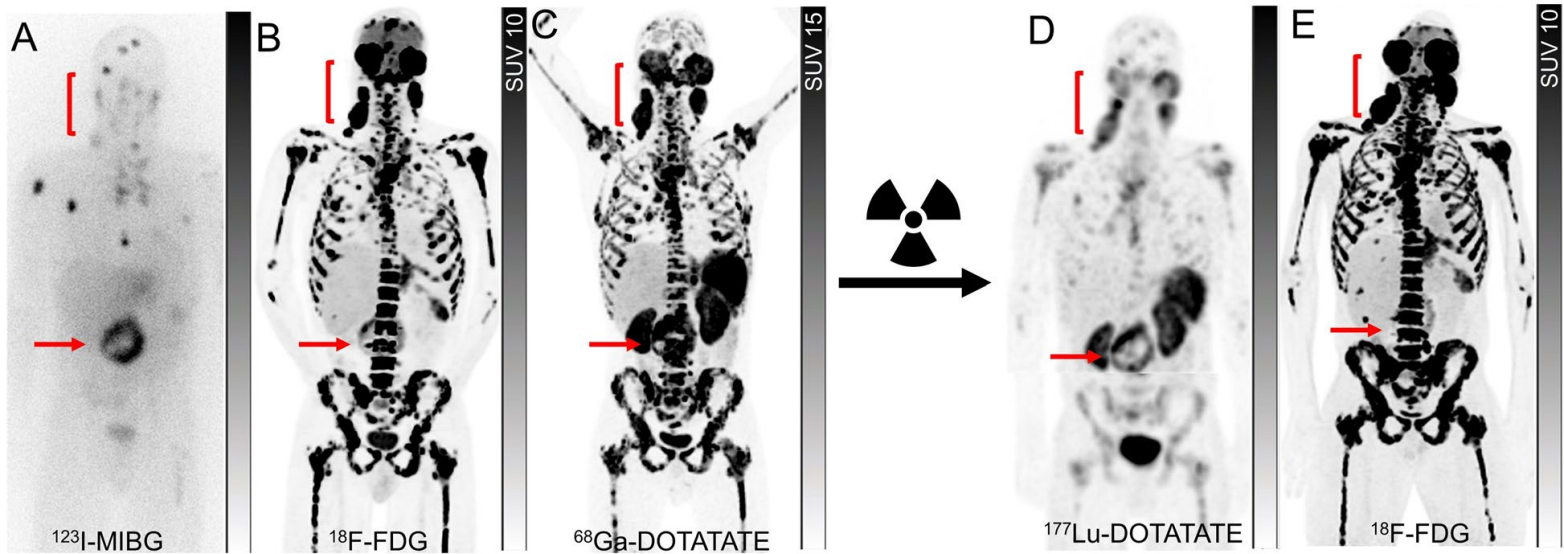
A patient with the organ of Zuckerkandl metastatic paraganglioma with progression on three lines of chemotherapy, three lines of targeted therapies and immunotherapy was referred for radionuclide therapy. ^123^I-MIBG planar imaging (**A**) showed intense uptake in the organ of Zuckerkandl (arrows) and a limited number of bone metastases compared to far more extensive bone metastases and lymphadenopathy in the bilateral posterior neck (brackets) on a contemporaneous ^18^F-FDG PET (**B**). Therefore, the patient was not considered suitable for treatment with ^131^I-MIBG. ^68^Ga-DOTATATE PET (**C**) showed high tracer avidity (Krenning score 4) in most sites of disease similar in distribution to ^18^F-FDG PET, hence the patient was deemed suitable for ^177^Lu-DOTATATE. The post-treatment ^177^Lu-DOTATATE SPECT (**D**) showed moderate to intense uptake in the primary site and lymphadenopathy while the vast majority of bone metastases retain only mild to moderate tracer uptake (Krenning score 2–3). The patient developed progressive pancytopenia, which combined with the low post-treatment radiotracer uptake, did not advance with further cycles of ^177^Lu-DOTATATE. ^18^F-FDG PET (**E**) 2 months post-^177^Lu-DOTATATE showed further progression of the disease

Pheochromocytoma and paraganglioma (PPGLs) are NENs arising from the chromaffin cells of the adrenal medulla or extra-adrenal paraganglia, respectively. PPGLs are becoming an archetypical example of precision medicine, where the germline or somatic mutation determines the choice of diagnostic and treatment [41, 42]. About 70% of the PPGLs can be classified into 3 genomic clusters, each with distinct clinical behavior, prognosis, biochemical presentation, and molecular imaging diagnostic [42]. These include pseudohypoxia-related clusters 1A and 1B; kinase signaling–related cluster 2; and Wnt signaling–related cluster 3 [42]. The advances in the molecular imaging of PPGLs have led to a diverse range of potential imaging radiopharmaceuticals, each targeting a distinct cellular uptake mechanism, including SSTR (^111^In octreotide scintigraphy or SSTR PET), noradrenaline transporters (^123^I-MIBG, ^124^I-MIBG and ^18^F-MFBG), L-type amino acid transporters (^18^F-DOPA) and GLUT transporters (^18^F-FDG) [43, 44]. The choice of diagnostic molecular imaging can be guided by the cluster. For instance, the most sensitive molecular imaging modality for cluster 1A with SDHx mutation is SSTR PET [45, 46]. In a prospective series of 17 patients with SDH-B mutated metastatic PPGLs, SSTR PET imaging with ^68^Ga-DOTATATE had a lesion-based detection rate of 98.6%, higher than that of ^18^F-FDG PET/CT (85.8%), and ^18^F-DOPA PET/CT (61.4%) [46]. The patients with germline SDH-B mutation (~ 40% of all patients with metastatic PPGLs) frequently have a negative ^123^I-MIBG scan and thus are not amenable to ^131^I-MIBG therapy (Fig. 7) [47, 48]. MIBG, however, remains a relevant target in the detection and treatment of metastatic pheochromocytomas [49, 50].

**Fig. 8:**
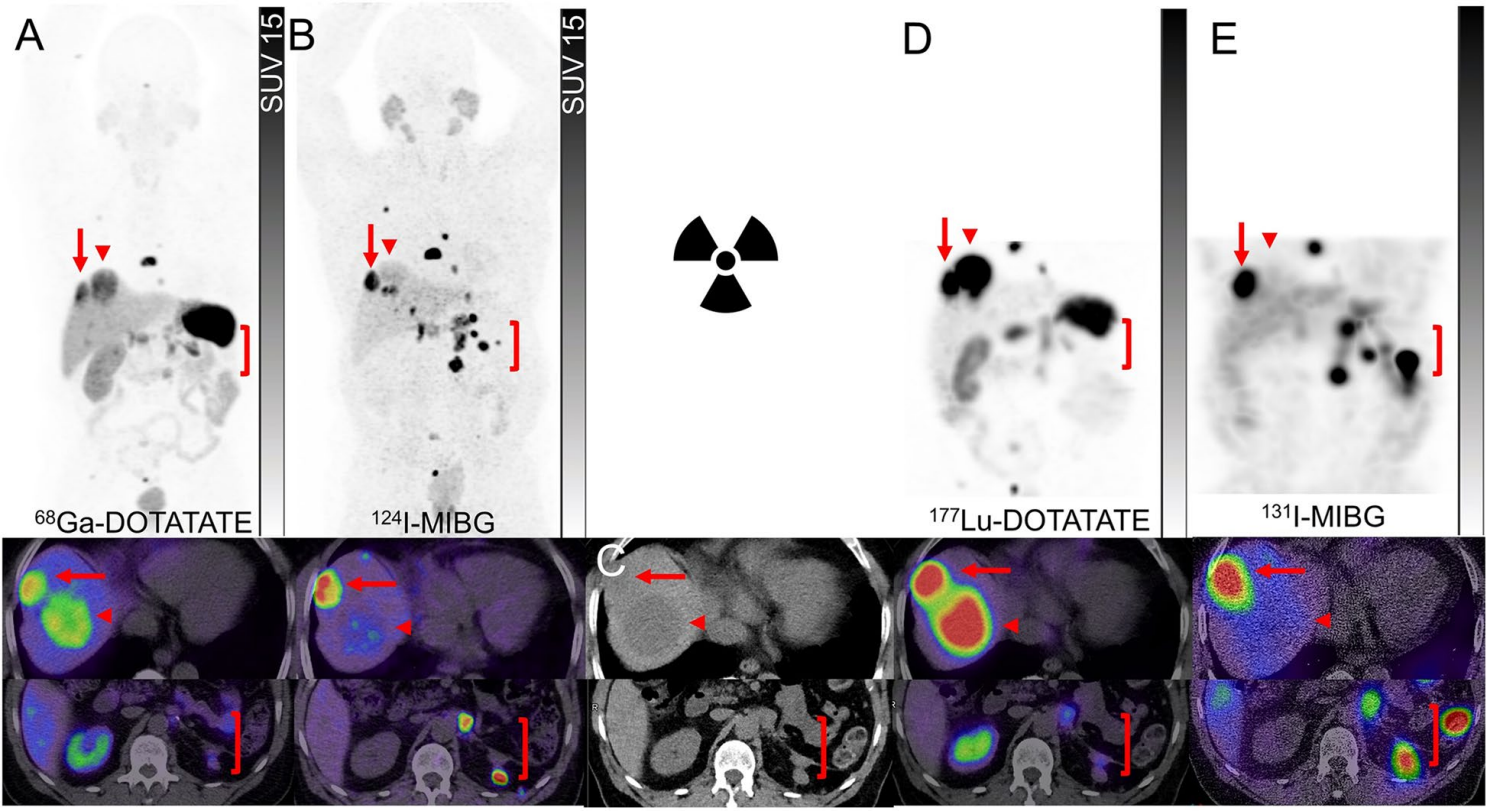
A patient with metastatic pheochromocytoma, previously treated with left adrenalectomy, presented with rapidly rising metanephrines. ^68^Ga-DOTATATE PET and PET/CT(**A**) showed intense uptake (Krenning score 4) in a thoracic vertebra, two dominant moderately avid (Krenning score 3) hepatic dome lesions (arrows and arrowheads), and mild to moderately avid soft tissue recurrence in the left upper quadrant surgical bed (brackets). ^124^I-MIBG PET and PET/CT (**B**) showed intense uptake in the bone metastases, variable uptake in hepatic dome lesions [a lesion with mild uptake (arrowheads) and other with intense uptake (arrows)], and intense uptake in the left upper quadrant soft tissue lesions (brackets). Corresponding CT images showed hypodense lesions in the liver (arrow and arrowhead) and soft tissue nodules in the left upper quadrant (bracket) (**C**). Due to heterogeneity of tracer uptake on both pre-treatment PET scans and the inability to target all sites of disease using a single therapeutic radiopharmaceutical, the patient underwent tandem treatment with 8 GBq of ^177^Lu-DOTATATE and 2.7 GBq of ^131^I-MIBG. Post-treatment ^177^Lu-DOTATATE SPECT and SPECT/CT (**D**) showed intense uptake in the bone and both hepatic dome metastases (Krenning score 4) (arrows and arrowheads) but only mild uptake in left upper abdomen soft tissue lesions (brackets). Similar to pre-treatment ^124^I-MIBG PET, post-treatment ^131^I-MIBG SPECT and SPEC/CT (**E**) demonstrated intense uptake in the bone, intense uptake in abdominal left upper quadrant soft tissue lesions (brackets) but in only one of the hepatic lesions (arrows) with no significant uptake in the other (arrowheads), emphasizing the ability to cover disparate molecular imaging phenotypes by using a combination of therapeutic radiopharmaceuticals

While surgical resection remains the backbone of the majority of cases, for non-surgical or metastatic disease, genetically driven cluster-specific therapy appears to be appealing but not widely established in routine clinical practice [42]. A unique opportunity has been provided to nuclear medicine for personalized treatment by the availability of two radionuclide therapy agents, ^131^I-MIBG and ^177^Lu-DOTATATE. Molecular imaging with ^123^I/^124^I-MIBG and SSTR PET can serve as the primary gatekeeper for the assessment of the most suitable radiopharmaceutical therapy for the patients [51]. Despite the lack of comparative studies, the expert consensus opinion recommends prioritizing the choice of radiopharmaceutical therapy based on the agent with the highest tracer uptake on pre-treatment imaging (Figs. 7 and 8) [51, 52]. Some additional considerations include the distinct toxicity profiles of these agents, relevant risk factors for developing toxicity and variable availabilities of the diagnostic and therapeutic pair across the world. It is also noteworthy that at more advanced stages of the disease, there may be a role for ^18^F-FDG PET to detect sites of dedifferentiated disease which may be non-targetable by one or either of these radionuclide therapy agents, however, this needs to be further verified in future trials [53].

## Other cancers with neuroendocrine features

### Thyroid carcinoma

Radioiodine therapy with ^131^I-sodium iodide forms the mainstay in the treatment of advanced differentiated thyroid carcinoma (DTC) after surgery and is the first-line therapy for metastatic DTC. However, approximately 20–30% of patients with DTC are refractory to radioiodine or lack sufficient uptake to warrant treatment and, therefore, require alternative therapies. Variable levels of SSTR expression have been reported in DTC. However, the relatively uncommon and aggressive Hürthle cell carcinoma has a relatively higher SSTR expression compared to the more common papillary and follicular carcinomas [54]. ^177^Lu-DOTATATE therapy performed in a small series of five patients with DTC showed slightly better outcomes in patients with Hürthle cell carcinoma  compared to papillary and follicular thyroid carcinoma [55]. The sub-optimal results of SSTR targeted imaging and therapy in DTC likely stem from the variable and relatively low-grade SSTR expression on the tumour cells. Patient selection using the modified Krenning score renders only a small fraction of patients with radioiodine-refractory DTC that would be eligible for ^177^Lu-DOTATATE therapy. Other radiotracers, including the neo-angiogenesis targeting ^68^Ga-RGD might be better suited for imaging and evolving theranostics [56, 57].

Medullary thyroid carcinoma (MTC) is a neuroendocrine malignancy originating from the parafollicular C-cells. It poses a challenge, both in terms of detection and treatment, as about 50% of patients experience disease recurrence/ relapse despite aggressive primary surgical treatment [58]. SSTR targeting has been of utility in both the diagnosis and treatment of patients with MTC [44, 45, 59]. A prospective study of 31 patients with metastatic MTC treated with ^90^Y-DOTATOC showed no correlation between the grade of radiotracer avidity on SSTR imaging (planar ^111^In-pentetreotide scintigraphy) and response to treatment [60]. However, it must be noted that over 50% of patients in this cohort had a radiotracer uptake lower than that of the liver (corresponding to Krenning score 1 or 2), with diffusely metastatic disease and no avidity on ^18^F-FDG PET. It is quite plausible that the majority of these participants might have harbored aggressive disease at baseline, not amenable to PRRT. A retrospective review of 43 patients with metastatic MTC who underwent ^177^Lu-DOTATATE therapy showed lesion size (< 2 cm) and low avidity on ^18^F-FDG PET (SUVmax < 5) to be significant predictors for treatment response [61]. ^18^F-DOPA and ^18^F-FDG have been shown to provide complementary information in the re-staging of patients with MTC. Similar to GEP NENs, ^18^F-FDG positivity is associated with aggressive tumour biology, reflected by rapidly doubling times for serum calcitonin. On the other hand, ^18^F-DOPA PET detects the less-aggressive tumours, often with lower calcitonin levels and longer calcitonin doubling times [62]. Molecular imaging of MTC remains challenging and the role of other novel peptide receptors that might be suitable for the treatment of MTC is discussed later in this manuscript.

### Merkel cell carcinoma

**Fig. 9:**
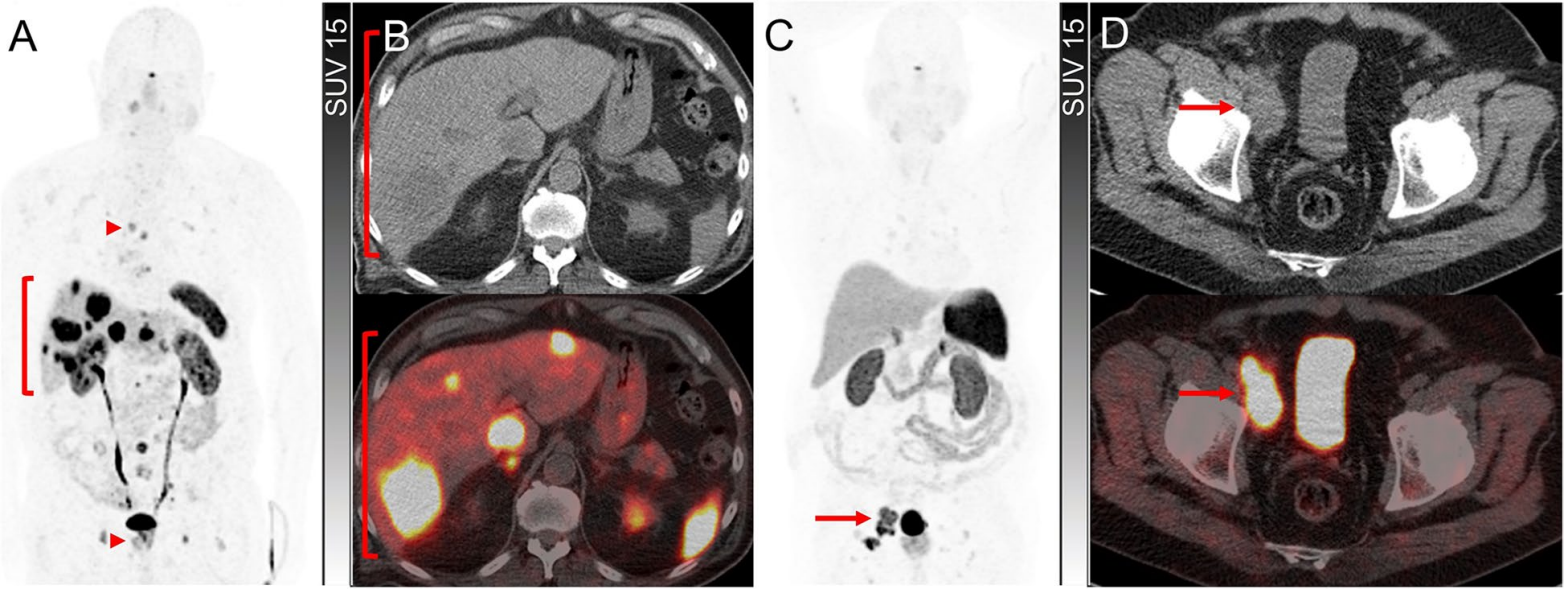
A patient with prior history of adenocarcinoma of the prostate with rapidly progressive obstructive uropathy with multiple progressive liver lesions. The biopsy of the prostate and a liver lesion was consistent with neuroendocrine differentiation of prostate cancer. ^68^Ga-DOTATATE PET (**A**), CT and PET/CT (**B**) showed multiple intensely tracer avid hepatic lesions (brackets) with lower activity in the prostate and scattered bone metastases (arrowheads). A patient with Merkel cell carcinoma with progression on multiple lines of therapy including immunotherapy, chemotherapy, targeted therapy with ^68^Ga-DOTATATE PET (**C**), CT and PET/CT (**D**) showing intensely tracer avid lymphadenopathy in the right external iliac and inguinal regions (arrows)

Merkel cell carcinoma is a rare and highly aggressive cutaneous neuroendocrine tumour. Immune checkpoint monoclonal antibodies have replaced chemotherapy as the frontline treatment with durable response in half of the patients [63]. SSTR-2 immunohistochemical assessment of 98 patients demonstrated heterogeneity of expression with low and moderate staining scores equally in approximately 30% of patients but none with strong staining scores [64]. Imaging with SSTR PET has shown generally high sensitivity in detecting disease which has been comparable to ^18^F-FDG PET in a small study [65]. The clinical experience with SSTR-2 based PRRT remains limited to several case reports [66, 67]. However, it remains to be defined what proportion of patients will have sufficient uptake on pre-treatment SSTR PET to warrant consideration of ^177^Lu-DOTATATE alone or in combination with immune checkpoint monoclonal antibodies. A phase Ib/II study (NCT04261855) is underway that will evaluate the efficacy of the combination of avelumab with ^177^Lu-DOTATATE (Fig. 9).

### Neuroendocrine prostate carcinoma

Histological classification of neuroendocrine prostate cancer (NEPC) has undergone multiple refinements. These include a spectrum of presentations extending from neuroendocrine differentiation in prostate adenocarcinoma, mixed with acinar adenocarcinoma, carcinoid tumour to small cell carcinoma [68]. In its most aggressive form, it is characterized by treatment-induced androgen receptor-independent prostate cancer, which appears to be a significant part of the broader category of castration-resistant tumours. The NE differentiation markers including synaptophysin, chromogranin, and CD56 need to be present for diagnostic confirmation [69]. Platinum-based chemotherapy typically results in short-lived responses with the generally dismal outcome; hence, novel diagnostic and therapeutic strategies are critically needed to improve patient outcomes. Similar to other high-grade neuroendocrine tumours/carcinoma, ^18^F-FDG has demonstrated clinical utility in detecting NEPC [70]. While targeting the prostate-specific membrane antigen (PSMA) has been increasingly established in the diagnosis and treatment of prostate adenocarcinoma, preclinical and early clinical studies are suggesting its limited utility in NEPC. The patient-derived xenograft models are indicative of suppression of FOLH1 gene (which encodes PSMA expression) with amplification of SSTR-2 expression in NEPC, making the latter a potential target for imaging and treatment [71]. Studies with a limited number of patients have suggested SSTR PET avidity of metastatic sites in half of the patients [72]. However, given inter and intra-lesional heterogeneity of tracer avidity on SSTR PET, it remains to be determined whether SSTR-2 based PRRT alone or in combination with other therapeutic strategies is a viable treatment option (Fig. 9) [73–75].

## Novel agents and alternative peptide receptors for molecular imaging and therapy

### Somatostatin antagonists

Radiolabeled SSTR antagonists are one of the different strategies aimed at improving the targeting of this peptide receptor. Despite a low internalization rate, SSTR antagonists have greater receptor binding sites due to the recruitment of inactive SSTRs on the cell surface of NENs. The three SSTR antagonists of clinical interest include LM3, JR10 and JR11 and have been conjugated to the chelators DOTA and NODAGA and complexed with various diagnostic radiometals such as ^111^In, ^64^Cu and ^68^Ga and therapeutically to ^177^Lu [76]. NODAGA appears to be the chelator of choice due to the significantly higher affinity of ^68^Ga-NODAGA-JR11 compared to ^68^Ga-DOTA-JR11 [76]. Interestingly, in this study, while ^68^Ga-DOTA-JR11 detected significantly more liver lesions, due to lower liver uptake, it showed fewer bone lesions compared to ^68^Ga-DOTATATE [77]. It appears ^68^Ga-NODAGA-JR11 is the preferred diagnostic SSTR antagonist and can be used as theranostic pair of ^177^Lu-DOTA-JR11. A first-in-human study of the theranostic pair of ^68^Ga-NODAGA-LM3 and ^177^Lu-DOTA-LM3 has shown a favorable biodistribution, efficacy and toxicity profile with a longer effective half-life in tumours and normal organs leading to higher radiation doses than SSTR agonists [78].

### Novel peptide receptors

In addition to SSTR-2 and SSTR subtypes (SSTR-3 and SSTR-5), other regulatory peptide receptors such as incretin receptor glucagon-like peptide 1 (GLP-1) and cholecystokinin receptor family (CCK-1 and CCK-2 subtypes), glucose-dependent insulinotropic polypeptide receptor (GIP) and gastrin-releasing peptide receptors are overexpressed in several human cancers [76]. GLP-1, CCK-2 and GIP receptors are of interest in NENs with concomitant overexpression having been identified even in a single sample [6]. The endogenous peptides for these regulatory receptors are potent and of low molecular weight, which allows rapid penetration into the tissues with specific binding. These properties can be leveraged by radiolabeling peptides for diagnosis and potential therapeutic purposes.

**Fig. 10:**
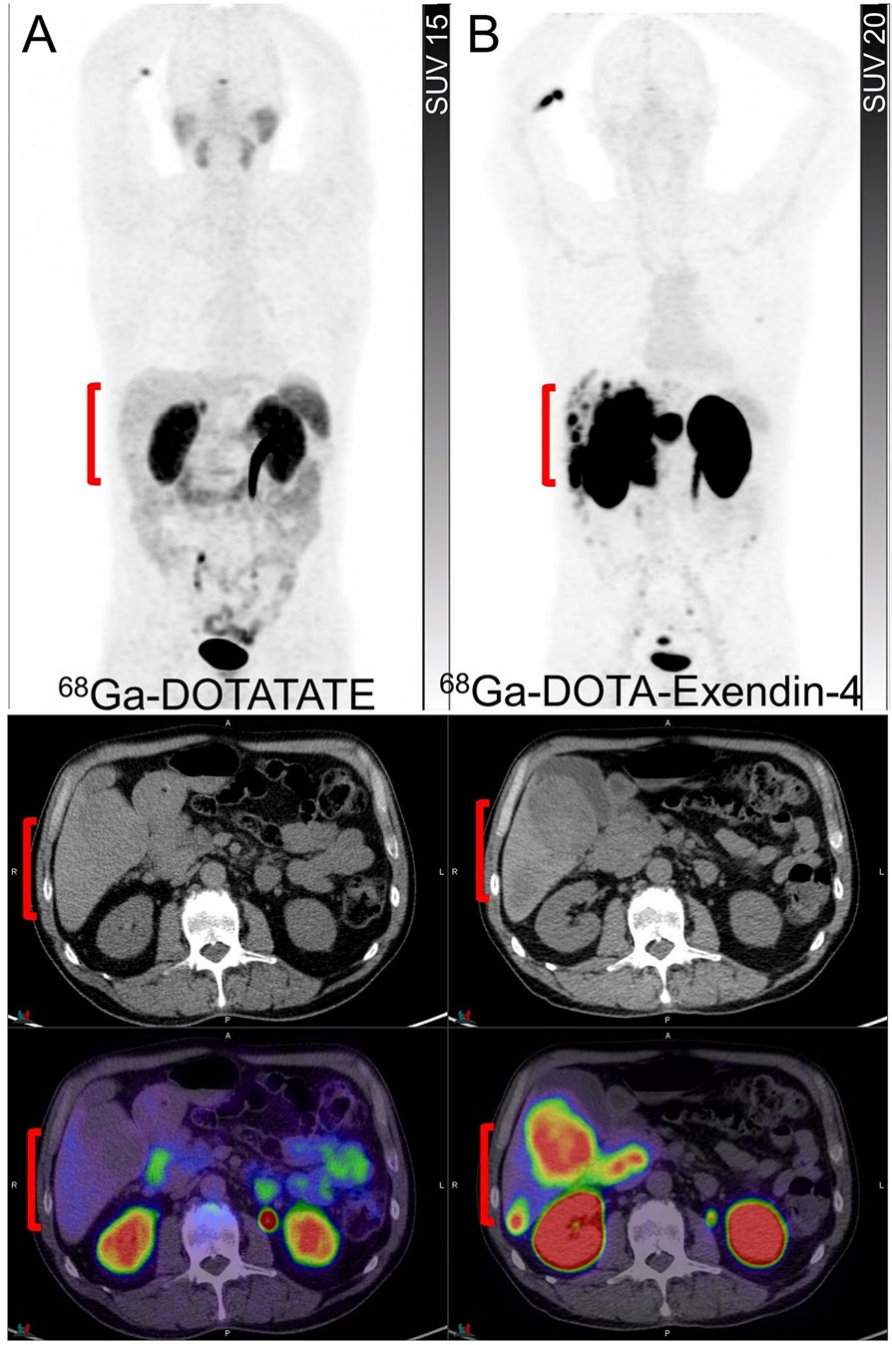
A patient with well-differentiated duodenal NET (G1, Ki67 < 2%) underwent ^68^Ga-DOTATATE for the assessment of suitability for ^177^Lu-DOTATATE. ^68^Ga-DOTATATE PET CT and PET/CT (**A**) showed no uptake in the hepatic metastases (brackets). ^68^Ga-DOTA-Exendin-4 PET, CT and PET/CT (**B**) showed intense uptake in the hepatic metastases (brackets)

GLP-1 receptors are widespread within the gastrointestinal tract and pancreas, and overexpressed in various NETs (Fig. 10), particularly in high density in almost all benign insulinomas [79]. Exendin-4 is a long-acting GLP-1 analog used for the treatment of type 2 diabetes and as the precursor to be radiolabeled with a variety of gamma-emitting radionuclides including ^111^In and ^99m^Tc, as well as PET radionuclides such as ^68^Ga, ^64^Cu, ^18^F and ^89^Zr. PET tracers have a higher special resolution compared to gamma-emitting tracers, which is a major advantage in the detection of small-sized insulinomas. The two most used tracers in clinical studies include ^68^Ga-DOTA-exendin-4 and ^68^Ga-NODAGA-exendin-4 [80, 81]. Insulinoma is a usually benign, insulin-producing NEN involving the beta-islet cells of the pancreas with the clinical symptoms typically resulting from the endogenous hyperinsulinemic hypoglycemic state. Their small size and variable location in the pancreas limit detection on conventional imaging such as contrast-enhanced CT [82]. In contrast with the NENs of other sites, benign insulinomas have a relatively lower expression of SSTRs [6]. ^68^Ga-NODAGA-Exendin-4 PET has shown a significantly higher sensitivity compared to SSTR PET (93.5% vs 61.3%, respectively) and appears to be superior to conventional imaging for the detection and localization of benign insulinoma [83]. This strategy may also help improve the detection of small lesions in the tail of the pancreas that can be obscured by high uptake in the adjacent left kidney [84]. A prospective trial is assessing the accuracy of ^68^Ga-NODAGA-exendin-4 PET in the detection of insulinoma compared to conventional imaging (NCT03189953). The proposed clinical utility of this agent includes localization of potentially resectable insulinoma for the work-up of endogenous hyperinsulinemic hypoglycemia following inconclusive conventional imaging [85]. However, the efficacy of this agent for the exclusion of cases of suspected nesidioblastosis and post-gastric bypass hypoglycemia is questionable [86]. Although ^18^F-DOPA has demonstrated high localization rates for insulin-producing tumours, especially in the pediatric population [8], in a small study, using a post-resection sample as the standard of truth, ^68^Ga-NODAGA-exendin-4 showed a higher target-to-background, interobserver agreement and higher sensitivity (100% versus 71%) in the detection of focal abnormality in congenital hyperinsulinemia compared to ^18^F-DOPA PET [81]. Although exendin-4 has been successfully radiolabeled with ^111^In and ^177^Lu, preclinical studies indicate a prohibitive absorbed dose to kidneys, limiting the therapeutic index of exendin-based PRRT [87]. Further research is required in the use of strategies to reduce renal absorbed dose such as renal protective agents. Around 10% of insulinomas are malignant and have a different spectrum of receptor expression [88]. In contrast to benign insulinoma, malignant or metastatic insulinoma often lack GLP-1 receptor while overexpressing SSTR-2, making them potentially suitable for SSTR-2 based PRRT [85, 89].

The CCK-2/gastrin receptor is overexpressed in several tumour types, including NENs, such as MTC (> 90%), GEP NETs (> 20%, except insulinoma which is higher), and approximately two-thirds of bronchial NET but also other tumours such as small cell lung cancer [6, 90]. The expression of SSTR is significantly lower than the CCK-2 receptor in MTC, making it a suitable target for imaging and therapy [91]. Among several peptides, DOTA-CP04 showed the most promising characteristics with high in-vivo stability, receptor affinity, high and prolonged tumour uptake against low renal retention. This agent can be labeled with ^111^In which is currently in a clinical trial [92] and also PET radioisotope ^68^Ga [93]. Given the more heterogenous expression of SSTR-2 in bronchial carcinoids than GEP NETs, DOTA-CP04 may have a role in a subset of these patients (Fig. 5) [14]. Radiolabeling of CCK-2/gastrin analogs with ^177^Lu has been successful in preclinical studies [94]. However, the field of CCK-2 receptors diagnostic and therapeutic is evolving and clinical success is still awaiting [95].

The gastric inhibitory polypeptide (GIP) receptor is a promising target with high incidence and receptor density expression in a variety of GEP and bronchial NENs and low expression in normal tissues [76]. GIP receptor targeting radiotracers can have a complementary role in molecular imaging of NENs as high overexpression of GIP receptor has been found in the majority of SSTR negative and GLP-1 negative NENs [96]. Several analogs have been developed and the preclinical studies have shown the specific tumour visualization by ^111^In-DTPA -GIP or ^68^Ga–DOTA-GIP [97, 98]. Theranostic clinical data on targeting GIP receptors are awaited.

In-vitro studies have demonstrated NENs often coexpress more than one and often up to 3 peptide receptors [99]. The ability to image multiple targets simultaneously through heterobivalent or heteromultivalent ligands appears a promising approach to overcome the target expression heterogeneity [100]. While sequential administration of radiopeptides is currently the most practical approach, the co-administration of a cocktail of radiopeptides is an alternative approach that may permit more efficient targeting of the tumours without the risk of stunning, which may limit the efficacy of subsequent administrations when more than one receptor is present. These approaches allow optimizing the in-vivo targeting of disease by selecting the most suitable radiopeptide(s) for the diagnosis and treatment.

## On-treatment molecular imaging phenotyping: qualitative assessment and dynamic risk stratification

Monitoring of biological target expression during the targeted treatment is desirable. Gamma decay of ^177^Lu allows for post-treatment whole body mapping of the radiopharmaceutical distribution and the relative intensity uptake ratio of tumour lesions to normal organs provides important information on the therapeutic index of ^177^Lu-DOTATATE. The quantitative analysis of post-treatment images in its most sophisticated method by SPECT at multiple time points from 1 h to 7 days yields a radiation dose map to the tumour and normal organs. However, the lack of consistent and uniform dosimetry methodology, limited accessibility due to its resource-intensive nature, patient burden requiring additional clinic visits and lack of reimbursement in some jurisdictions have posed significant challenges in its wide adoption by the institutions across the world. Meanwhile more simplified dosimetry methods are actively pursued. In addition to quantitative assessment, the relevant information obtained from a qualitative assessment of post-treatment SPECT/CT imaging may impact the patient management and dynamic risk stratification in a subset of patients.

**Fig. 11:**
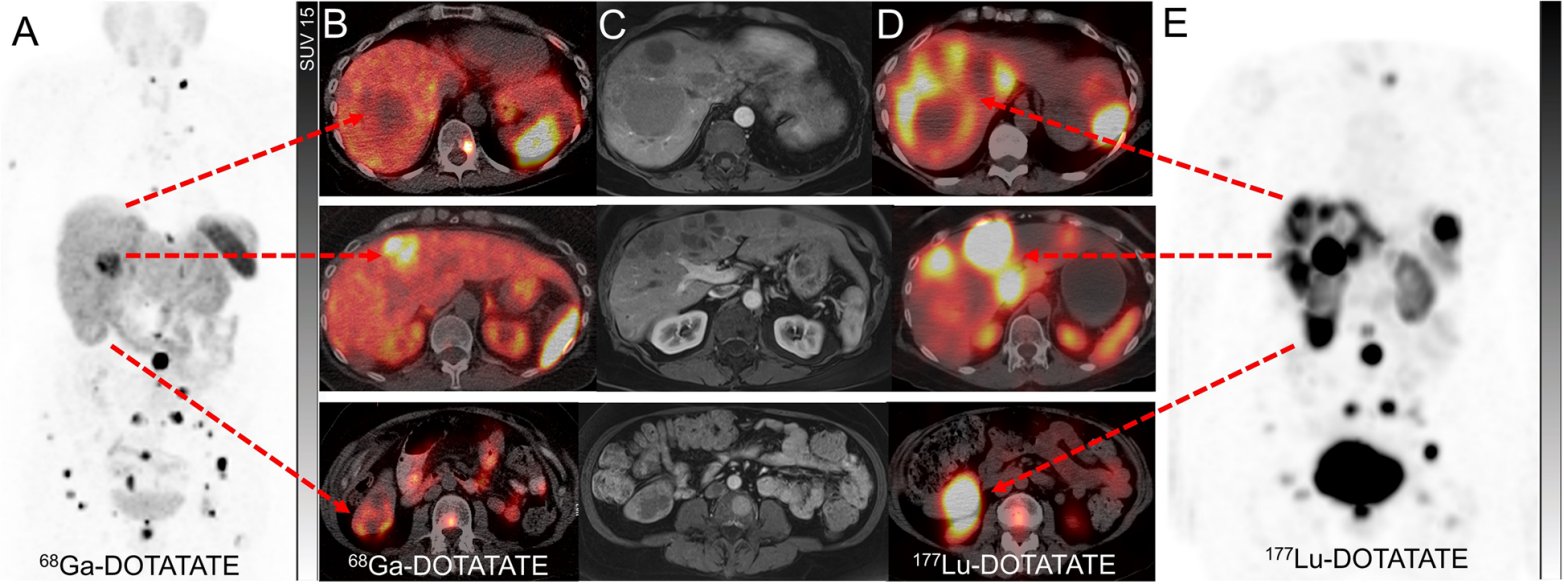
A patient with rectal NET (G2, Ki 67 12%) with progressive disease despite long-acting somatostatin analogues underwent ^68^Ga-DOTATATE PET (**A**), which showed intense uptake in the mesorectal lymph nodes and bone lesions but sufficient uptake only in one of the hepatic metastases (Krenning score 3–4) on PET/CT (**B**), while other contrast-enhancing lesions on MRI (arterial phase, **C**) had uptake similar to background liver (Krenning score 2). Contemporaneous ^18^F-FDG PET did not show any avid disease (not shown). Following discussion at the NET multidisciplinary team meeting, the decision was made to proceed with ^177^Lu-DOTATATE but only proceed with further cycles if high tracer retention is noted on post-treatment SPECT imaging. Post-treatment ^177^Lu-DOTATATE SPECT/CT (**D**) and SPECT (**E**) imaging showed high tracer retention (Krenning score 4) in all hepatic lesions, including lesions that showed low ^68^Ga-DOTATATE. The patient proceeded with 4 cycles of ^177^Lu-DOTATATE

Pre-treatment SSTR PET is generally used to assess the eligibility for ^177^Lu-DOTATATE. The semiquantitative parameters and heterogeneity of the SSTR expression at baseline SSTR PET appear to predict the response and the outcome of ^177^Lu-DOTATATE [19, 101]. However, a wide overlap of these parameters between responders and non-responders limits their clinical utility in isolation to guide patient management [101]. Although it is generally accepted that the pre-treatment SSTR PET correlates closely with post-treatment ^177^Lu-DOTATATE scintigraphy imaging, variation between the lesional correlation of the theranostic pair can be encountered. These could be due to lesion heterogeneity and differences in target densities, as well as disrupted or increased regional blood supply by pathologic tumour vessels that may affect the rate of uptake and retention of the radiotracer in the tumours. In addition, differing SSTR-2 affinity of ^68^Ga-DOTATATE (IC50 = 0.2 nM) compared to ^177^Lu-DOTATATE (IC50 = 2.0 nM) may need to be considered [102]. Specific clinical scenarios when post-treatment imaging can guide the management may include cases when the therapeutic index of treatment is in doubt, for instance, borderline tracer uptake in all lesions or significant inter- or intra-lesion heterogeneity of SSTR expression on pre-treatment SSTR PET (Figs. 6, 7, 8 and 11). Post-^177^Lu-DOTATATE scintigraphy can guide the decision of either completing the standard course of treatment (4 cycles) for instance in the context of emerging toxicities, or early abortion due to futility (Fig. 7). Other examples include patients with a highly responsive disease, usually of higher grade/ radiosensitive disease, when the number of cycles of treatment can be abbreviated upon marked response with loss of target on post-treatment scintigraphy images and the remainder of cycles potentially be deferred to future progression.

## Post-treatment molecular imaging phenotyping: ongoing dynamic risk stratification

**Fig. 12:**
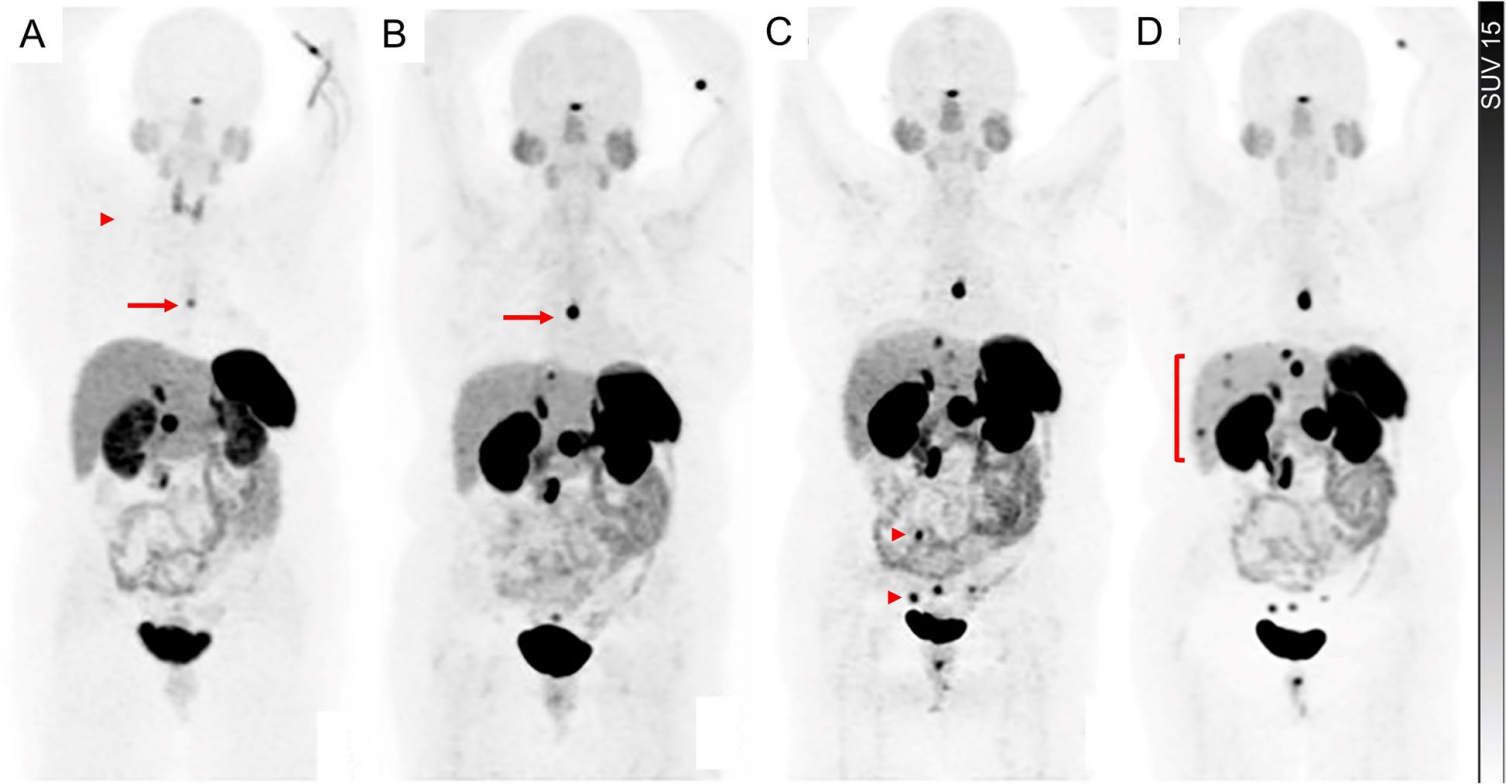
A patient with small intestinal NET (G1, Ki67 2%) with serial ^68^Ga-DOTATATE PET images performed at 6-monthly intervals for disease surveillance was commenced on long-acting somatostatin analog therapy between the base-line scan (**A**) and 6-month scan, as indicated by the loss of thyroidal uptake (arrowhead) between these scans. Despite the possibility of “pseudoprogression” related to altered biodistribution following the introduction of SSAs, asymptomatic status and relatively stable measurable lesions on CT, rising chromogranin-A levels suggested progressive disease, which was confirmed by increasing intensity of metastasis in the thoracic region between the baseline (**A**, arrow) and 6-month (**B**, arrow) scans, and progression of peritoneal metastases in the pelvis between the 6- and 12-month (**C**, arrowheads) scans and an increasing number of liver lesions, most apparent on the 24-month scan (**D**, bracket)

Although response monitoring by contrast-enhanced CT or MRI is the mainstay of the treatment response, the known limitations include assessment of the small volume lymph nodes, reproducibly measuring large volume coalescing hepatic lesions, variable conspicuity of the lesions due to differences in the timing of contrast administration and difficulty in response assessment of cystic/necrotic/hemorrhagic lesions [103]. Furthermore, given the indolent nature of well-differentiated NENs, RECIST criteria are not well suited for early detection of response or progression, especially after biologic or targeted therapies with expected minimal effect on tumour volume (Fig. 12).

A small number of studies including a limited number of patients have investigated the potential role of SSTR PET in the monitoring of the response after PRRT [104–108]. These studies have used various methods, including using the temporal change in the functional size or volume of the lesion based on SSTR PET or change in the intensity of uptake either qualitatively by Krennig score or semiquantitatively by absolute SUV values or its ratios to normal organs (liver or spleen). The studies have shown conflicting results in the ability of SSTR in predicting the outcome and none have been externally validated. However, important lessons have been learned from these studies.

Firstly, there is no consistent correlation between SUV changes and outcomes [104, 105]. Spatial (inter-and intra-lesional) and temporal heterogeneity of SSTR expression, as well as differing responsiveness of variably SSTR-expressing disease to treatment, may lead to dissociated responses between morphologic imaging and SSTR PET in some cases (Fig. 3). Therefore, temporal changes in the intensity of SSTR PET tracer uptake need to be interpreted in conjunction with the corroborative anatomical changes on CT or MRI [109].

Secondly, in a subset of patients, new lesions could be detected earlier by SSTR PET than morphological imaging up to several months [107]. These include small lymph nodes and a limited volume of peritoneal disease. Furthermore, SSTR PET is the preferred option in specific scenarios such as monitoring of response in bone dominant disease where morphological imaging lacks sensitivity and specificity [110].

Finally, combining SSTR PET and ^18^F-FDG PET appears to be a promising approach and may provide a more holistic assessment of varied components of the disease. For example, resolution of prior FDG-avid disease can be reassuring in the context of stable or increasing intensity in SSTR-expressing disease sites (Fig. 3). In a study of 66 patients with NENs, SSTR PET and ^18^F-FDG PET at baseline, 3 months and 6–9 months were used for response monitoring after PRRT [111]. ^18^F-FDG PET was complementary to SSTR PET and strongly correlated with a higher risk of progressive disease. Earlier and deeper metabolic response of the ^18^F-FDG avid component of disease (presumably less differentiated and more responsive disease) may provide an early risk stratification (Figs. 2, 3, 4). Conversely, the impact of the presence of ^18^F-FDG-avid disease at completion of treatment on management may include more intense follow-up and treatment strategy. Spatially discordant ^18^F-FDG-avid/non-SSTR-avid disease, at any stage of treatment should raise the possibility of less differentiated disease, be considered for biopsy if feasible and guide the choice of systemic treatments in widespread metastases or local treatment in limited disease (Fig. 4). There is an unmet need in incorporating ^18^F-FDG PET in clinical trial design throughout the course of disease, from the patient selection for PRRT to the post-treatment risk stratification, response monitoring and sequencing of the treatment modalities.

Given the complexity and heterogeneity of NENs, the response to treatment by morphologic and molecular imaging should be considered complementary and integrated with individual clinical, quality-of-life, and biochemical assessments. An individualized approach needs to be taken, for instance, while the patients with the liver dominant disease may need to be better assessed by morphologic imaging (CT or MRI), bone disease can be more efficiently followed up by molecular imaging. Beyond response assessment, the unique advantages of molecular imaging may need to be leveraged by its incorporation into the clinical trials to guide the choice of treatment modality, to risk stratify patients following the treatment and for the intensity and duration of follow-up. Development of segmentation tools that allow assessment of the serial evolution of molecular imaging phenotypic subclones will improve global response assessment and help to establish patterns of response that warrant different management paradigms. For example, an increasing volume of FDG-avid, low SSTR-expressing disease may warrant a change to chemotherapy even in the context of a partial response on SSTR-expressing disease based on volumetric regression. Such tools will need to be cross-validated with other measures of response and with clinical outcomes. Artificial intelligence algorithms may help integrating an increasingly complex and deep combination of data relevant to patient outcomes in this diverse and heterogeneous malignancy.

## Conclusion

Molecular imaging has become the mainstay of the management of patients with NENs. While the patient selection based on molecular imaging phenotype remains integral to the success of the radioligand therapy, temporal variation in the expression of the biological target and the responsiveness of the different components of disease during and after treatment are among increasingly understood roles of various molecular imaging agents in the post-treatment setting. In certain diseases such as PPGLs, genotyping is now entered the clinical practice and may dictate the choice of the molecular imaging agent, however, whole-body molecular imaging phenotyping remains the key to the success of the targeted radioligand therapy. Promising advances have been made in leveraging the novel peptide receptors to overcome the heterogeneity of the established targets for diagnosis and targeted therapy of certain neuroendocrine tumours. Future advances may include novel approaches to enhance target engagement such as using SSTR antagonists or multitargeted radiopharmaceuticals.

## Data Availability

Not applicable.

## Abbreviations

CCK: Cholecystokinin
DOPA: Dihydroxyphenylalanine
DTC: Differentiated thyroid carcinoma
FDG: Fluorodeoxyglucose
GEP: Gastroenteropancreatic
GIP: Glucose-dependent insulinotropic polypeptide
GLP: Glucagon-like peptide
GLUT: Glucose transporter
IHC: Immunohistochemistry
MFBG: Meta-Fluorobenzylguanidine
MIBG: Meta-iodobenzylguanidine
MTC: Medullary thyroid cancer
NEC: Neuroendocrine carcinoma
NEN: Neuroendocrine neoplasm
NEPC: Neuroendocrine prostate cancer
NET: Neuroendocrine tumour
OS: Overall survival
PFS: Progression-free survival
PPGL: Pheochromocytoma and paraganglioma
PRRT: Peptide receptor radionuclide therapy
PSMA: Prostate-specific membrane antigen
SDH: Succinate dehydrogenase
SSA: Somatostatin analog
SSTR: Somatostatin receptor
SUVmax: Maximum standardised uptake value
WHO: World health organization
